# Editorial: Monitoring and Promoting Physical Activity and Physical Fitness in Children

**DOI:** 10.3389/fpubh.2021.633457

**Published:** 2021-02-10

**Authors:** Stevo Popovic, Hugo Sarmento, Yolanda Demetriou, Adilson Marques

**Affiliations:** ^1^Faculty for Sport and Physical Education, University of Montenegro, Niksic, Montenegro; ^2^Research Unit for Sport and Physical Activity, Faculty of Sport Sciences and Physical Education, University of Coimbra, Coimbra, Portugal; ^3^Faculty of Sports and Health Sciences, Technical University of Munich, Munich, Germany; ^4^CIPER, Faculty of Human Kinetics, University of Lisbon, Lisbon, Portugal

**Keywords:** sports, exercise, fitness, physical education, sedentary behavior

## Introduction

Health is an important life resource that each person has. The preservation of a good state of health is important for the realization of the personal objective of life that each person defines for himself. Thus, from an early age, the determinants of health were identified. Among the various determinants of health, physical activity, and exercise stood out from an early age. It quickly became apparent that the most physically active people had better health indicators. Susruta (1500 BC), an Indian physician, was the first who prescribe daily exercise, while Hippocrates (460–370 BC) was the first one who provides a written exercise prescription for a patient suffering from consumption. On the other hand, the importance of physical fitness is recorded in the conversation between Socrates and one of Socrates' disciples named Epigenes. On noticing his companion was in poor condition for a young man, the philosopher admonished him by saying, “You look as if you need exercise, Epigenes.” To which the young man replied, “Well, I'm not an athlete, Socrates.” Socrates then offered an answer that was remembered as an anthology (1).

Physical inactivity is the fourth leading cause of premature death (2) since it can cause chronic diseases, such as obesity, type II diabetes, hypertension, cardiovascular diseases, or colon and breast cancers (3). However, a high percentage of children and adolescents in industrialized countries lead a sedentary lifestyle (4). A study by Cooper et al. (5) analyzing pooled accelerometer data from more than 27,000 children and adolescents (aged 3–18) shows that only 9% of the male and 2% of the female participants meet the WHO recommendation of daily 60 min of moderate-to-vigorous physical activity. Concerning the development throughout childhood and adolescence, physical activity decreases on average by about 4% with each year of age after the age of six. Additionally, sedentary behavior, which nowadays is considered not just as the opposite of physical activity but to have its independent negative influence on health, is increasing (5, 6). Physical fitness is a multi-component construct and a health biomarker highly correlated to physical activity (7, 8).

In addition to the impact on health, it has been shown that physical activity and physical fitness may significantly improve academic performance. There is a body of evidence to support this claim (9). In the first place, it is important to point out that mathematics and reading are academic topics that are most influenced by physical activity, but also the fact that basic cognitive functions that facilitate learning such as attention and memory can be improved by physical activity and greater aerobic fitness. Given the importance of monitoring and promoting physical activity and physical fitness in children, mostly due to the reason the single sessions of long-term participation in physical activity improve cognitive performance and brain health, as well as the reason the children who participate in vigorous- or moderate-intensity physical activity benefit the most, this Research Topic was created.

## Contribution to the Field

The purpose of this Research Topic was to gather the latest knowledge in the field of monitoring and promoting physical activity and physical fitness in children. Several studies that emerged as the output of this special edition have pushed the boundaries of our knowledge, at least by so much that it is worth mentioning them and emphasizing that the whole work was meaningful and made a significant contribution to the scientific field.

Some interesting findings were reached in this Research Topic. First, an interesting finding regarding the evaluation of physical activity and physical fitness was that. Using physical fitness as a criterion to measure PA seems to be an effective option in terms of both the economic and organizational sense when reporting the monitoring, surveillance, and evaluation of PA interventions (Sember et al.). Additionally (Niessner et al.) presented a new LMS (least-mean-squares) coefficient to compare children's physical fitness levels and LMS curves that are available by year from age 4 to 17 years. A further significant contribution is the finding that shows growing inequality and polarization of the motor development of children (Potočnik et al.).

Some authors have confirmed that the declining trend of neuromotor fitness may have important implications for enjoyment and participation in physical activity, and thus for future health (Anselma et al.), which can significantly improve the participation of certain populations in daily physical activity, equally as an offer of extracurricular activities (Kuritz et al.). Another of the published articles in this special issue has a similar goal and emphasizes the importance of body image perception, anthropometric values, and physical condition to be physically active. Besides, it emphasizes the mediating role of physical self-perception for the development of physical activity (Sánchez-Miguel et al.). Kobel et al. confirm the positive impact of physical exercises on endurance performance in kindergarten children, but no other motor ability. Incorporating methods to develop agility and to improve resilience may lead to better outcomes when designing physical fitness programs to prevent or alleviate anxiety in children (Li et al.). Having siblings showed to be advantageous for general physical fitness in children (Rodrigues et al.) and standing desks provide an opportunity to reduce sedentary time during lessons and breaks at school (Sprengeler et al.). However, another studies raised several research questions such as if functionalized play can provide the pleasures of children's free play (Frahsa and Thiel), or school-based physical activity projects such as skipping hearts can have a long-term impact on health and health behavior (Baumgartner et al.). These issues will trigger a significant number of new clinical studies that should make new scientific progress toward new knowledge and new practices that will improve the field of monitoring and promotion of physical activity in children.

## Conclusion

Mounting research indicates that physical activity and physical fitness are associated with health benefits in children. High levels of physical activity and physical fitness, mainly cardiorespiratory fitness, are associated with better health-related biomarkers that may further influence adulthood health. In contrast, time spent in sedentary behavior is associated with negative health outcomes. Therefore, now, more than ever, understanding and developing strategies to promote physical activity behavior and to improve children's fitness levels are essential. These strategies can be developed in the school setting or indifferent contexts. For that reason, the purpose of this Research Topic was to collect high-quality research relating to the monitoring and promotion of physical activity and physical fitness in the pediatric population with special attention to novel intervention research in school, community-based, or sports settings to promote children's levels of physical activity and physical fitness, as well as the effects of physical activity on physical fitness in children and adolescents and correlational and survey studies examining these relationships.

## Author Contributions

SP drafted the Editorial. HS, YD, and AM revised and approved the final version. All authors contributed to the article and approved the submitted version.

## Conflict of Interest

The authors declare that the research was conducted in the absence of any commercial or financial relationships that could be construed as a potential conflict of interest.
